# NOTCH3, a crucial target of miR-491-5p/miR-875-5p, promotes gastric carcinogenesis by upregulating PHLDB2 expression and activating Akt pathway

**DOI:** 10.1038/s41388-020-01579-3

**Published:** 2021-01-15

**Authors:** Wei Kang, Jinglin Zhang, Tingting Huang, Yuhang Zhou, Chi Chun Wong, Ronald C. K. Chan, Yujuan Dong, Feng Wu, Bin Zhang, William K. K. Wu, Michael W. Y. Chan, Alfred S. L. Cheng, Jun Yu, Nathalie Wong, Kwok Wai Lo, Ka Fai To

**Affiliations:** 1 Department of Anatomical and Cellular Pathology, State Key Laboratory of Translational Oncology, Prince of Wales Hospital, The Chinese University of Hong Kong, Hong Kong SAR, PR China; 2 Institute of Digestive Disease, State Key Laboratory of Digestive Disease, The Chinese University of Hong Kong, Hong Kong SAR, PR China; 3 Li Ka Shing Institute of Health Science, Sir Y.K. Pao Cancer Center, The Chinese University of Hong Kong, Hong Kong SAR, PR China; 4 Department of Gastroenterology, The Affiliated Drum Tower Hospital of Nanjing University, Medical School, Nanjing, PR China; 5 Department of Anaesthesia and Intensive Care, The Chinese University of Hong Kong, Hong Kong SAR, PR China; 6 Department of Life Science, National Chung Cheng University, Chiayi, Taiwan; 7 School of Biomedical Sciences, The Chinese University of Hong Kong, Hong Kong SAR, PR China; 8 Department of Medicine and Therapeutics, The Chinese University of Hong Kong, Hong Kong SAR, PR China

**Keywords:** Gastric cancer, Tumour biomarkers

## Abstract

Aberrant Notch activation has been implicated in multiple malignancies and the identification of NOTCH receptors and related pathways is critical for targeted therapy. In this study, we aim to delineate the most prominent dysregulated NOTCH receptor and comprehensively reveal its deregulation in gastric cancer (GC). In the four Notch members, NOTCH3 was found uniformly upregulated and associated with poor clinical outcomes in multiple GC datasets. siRNA-mediated NOTCH3 knockdown demonstrated antitumor effects by suppressing cell proliferation, inhibiting monolayer formation, and impairing cell invasion abilities. Its depletion also induced early and late apoptosis. NOTCH3 was confirmed to be a direct target of two tumor suppressor microRNAs (miRNAs), namely miR-491-5p and miR-875-5p. The activation of NOTCH3 is partly due to the silence of these two miRNAs. Through RNA-seq profiling and functional validation, PHLDB2 was identified as a potent functional downstream modulator for NOTCH3 in gastric carcinogenesis. PHLDB2 expression demonstrated a positive correlation with NOTCH3, but was negatively correlated with miR-491-5p. Akt-mTOR was revealed as the downstream signaling of PHLDB2. The NOTCH3-PHLDB2-Akt co-activation was found in 33.7% GC patients and the activation of this axis predicted poor clinical outcome. GC cells treated with siNOTCH3, siPHLDB2, miR-491-5p, miR-875-5p, were more sensitive to Cisplatin and 5-FU. Taken together, the NOTCH3-PHLDB2-Akt cascade plays oncogenic role in gastric carcinogenesis and serves as a therapeutic target. Our study provided insights into Notch-mediated underlying molecular mechanisms and implied translational potential.

## Introduction

Gastric cancer (GC) is one of the most common malignancies worldwide and it is the 3rd leading cause of cancer-related death in men and 5th in women [1]. Many potential risk factors influence the development of GC, including *Helicobacter pylori* (*H. pylori*) infection, Epstein–Barr virus (EBV) infection, chronic gastritis, the diet, and some genetic alterations. Despite advances in diagnostic modalities and the development of molecular-targeted drugs in the clinic, the overall survival of GC patients remains poor. Multiple genetic and epigenetic alterations are involved in gastric carcinogenesis, such as the activation of oncogenes, inactivation of tumor suppressor genes, as well as mutations in genes involved in cell adhesion molecules and DNA mismatch repair [2]. Histologically, GC can be mainly classified into diffuse type and intestinal type according to Lauren’s classification [3]. Accumulating studies have focused on the investigation of molecular mechanisms underlying the pathogenesis of GC. Molecular classification on the basis of the Cancer Genome Atlas (TCGA) research network defined four subtypes of GC: EBV-associated tumors, microsatellite unstable tumors (MSI), genomically stable tumors (GS), and tumors with chromosomal instability (CIN) [4]. Achieving a detailed molecular landscape of GC pathogenesis is vital to improve patient survival for this complex malignancy.

Multiple signaling pathways, such as Wnt/β-catenin signaling, Notch signaling, Hedgehog signaling, Hippo pathway, NF-κB, and epidermal growth factor receptor, are involved in gastric tumorigenesis. Interfering with these aberrantly activated signaling pathways underlies the rational development of molecular-targeted therapies [5]. Notch signaling is a conserved and prominent intracellular pathway that has been found to be deregulated in GC, and it regulates cell proliferation, apoptosis, and differentiation. There are four Notch receptors and five Notch ligands in mammals [6]. Activation of this pathway depends on ligand-induced proteolytic cleavage of Notch receptor, which in turn releases of the intracellular domain of Notch (NICD). Subsequently, NICD is translocated into the nucleus and activates multiple transcriptional programs that control diverse cellular functions [7]. The expression of cyclooxygenase-2 (COX-2) is induced by Notch signaling through the binding of NICD to the COX-2 promoter, resulting in GC progression [8]. Conversely, Notch suppression by γ-secretase inhibitor (GSI) promotes mitotic arrest and apoptosis in GC cells. Notch pathway inhibition leads to the activation of PTEN, which induces G2/M cell cycle arrest and suppresses GC progression [9]. Furthermore, Notch signaling pathway is activated after *H. pylori* infection in GC. However, the expression and functional roles of Notch receptors, NOTCH1-4, in GC initiation and progression remain unclear, and the molecular mechanism underlying NOTCH3 activation is not well defined.

In addition, microRNAs (miRNAs) have been reported to abnormally expressed in multiple types of cancers [10] and their deregulation are strongly associated with cancer initiation and progression [11]. miRNAs are small noncoding nucleotides 18–25 nucleotides in length. Mechanically, they directly interact with 3′ untranslated regions (3′UTR) of mRNA to degrade targeted mRNA or inhibit translation. miRNAs may function as either oncogenes or tumor suppressors [12]. In tumor initiation and progression, dysregulated miRNAs have been shown to regulate cell proliferation, invasion, metastasis, and angiogenesis [13].

In the current study, we comprehensively elucidated the expression pattern of Notch receptors, characterized the NOTCH3 cascade and revealed the NOTCH3 regulation by miRNA in GC.

## Results

### NOTCH3 is upregulated and associated with poor survival in GC

**Fig. 1:**
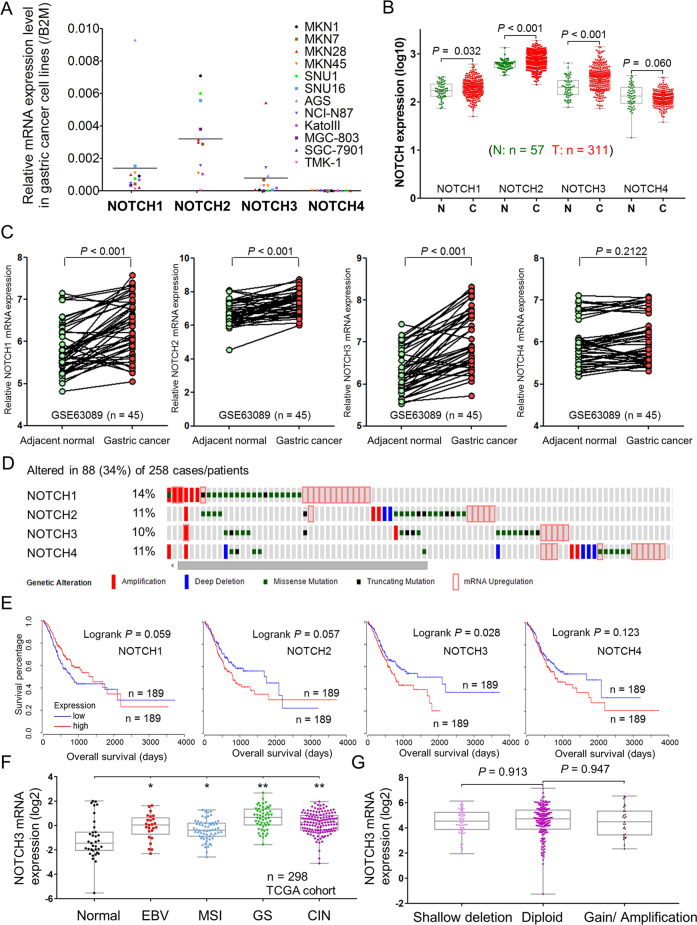
NOTCH3 is overexpressed in GC and correlated with poor survival. **A** NOTCH1-4 mRNA expression in 12 GC cell lines. **B** mRNA expression of NOTCH1-4 in primary gastric tumors and normal gastric epithelium samples from GENT cohort (NOTCH1, *P* = 0.032; NOTCH2, *P* < 0.001; NOTCH3, *P* < 0.001; NOTCH4, *P* = 0.060). **C** In 45 paired primary samples, NOTCH1-3, but not NOTCH4, is upregulated in gastric cancer samples compared with normal controls (NOTCH1, *P* < 0.001; NOTCH2, *P* < 0.001; NOTCH3, *P* < 0.001; NOTCH4, *P* = 0.2122; NCBI/GEO/GSE63089). **D** Genetic and epigenetic alteration rates of NOTCH1-4 among GC patients in TCGA cohort (*n* = 258). **E** Upregulated NOTCH3 indicated poor overall survival in TCGA cohort (*P* = 0.028). **F** The expression of NOTCH3 mRNA in the four molecular subtypes of GC and normal gastric samples (EBV EBV-positive, MSI microsatellite unstable, GS genomically stable, CIN chromosomal instability). **G** The correlation between NOTCH3 mRNA expression and its copy number aberrations (including shallow deletion, diploid, and gain/amplification).

NOTCH1-4 mRNA expression in 12 GC cell lines was evaluated. Quantitative real-time PCR (qRT-PCR) revealed that NOTCH4 has the lowest expression among the four Notch receptor members (Fig. 1A). In a cohort of primary GC tissues, NOTCH1-3 were highly expressed compared with normal gastric tissues according to GENT (Gene Expression across Normal and Tumor tissue) dataset (Fig. 1B). In another GC dataset, NCBI/GEO/GSE63089, NOTCH1-3 were also concordantly elevated in GC samples compared with paired non-tumorous tissues (Fig. 1C). Genetic and epigenetic alterations were then analyzed. Thirty-four percent of GC cases (88/258) have at least one alteration in *NOTCH1*-*4* from the cBioPortal database (Fig. 1D). These molecular alterations in *NOTCH1*-*4* include gene amplification (14%, 36/258), deletion (11%, 29/258), somatic mutation (10%, 25/258), and mRNA upregulation (11%, 29/258) [14]. In TCGA GC dataset, only NOTCH3 upregulation was significantly correlated with poor survival (Fig. 1E). Taken together, NOTCH3 was identified for further investigation because it is uniformly upregulated in multiple GC datasets. The high expression of NOTCH3 appeared to be independent of molecular subtypes, as the TCGA GC dataset demonstrated abundant NOTCH3 in all the four molecular subtypes (EBV, MSI, GS, and CIN), especially in GS GC (Fig. 1F) [14]. However, there was no positive correlation between NOTCH3 mRNA expression with its copy number variations (Fig. 1G), suggesting that transcriptional or post-transcriptional regulation might be responsible for its upregulation in gastric carcinogenesis.

### NOTCH3 knockdown exerts anti-oncogenic effect and promotes cell apoptosis

**Fig. 2:**
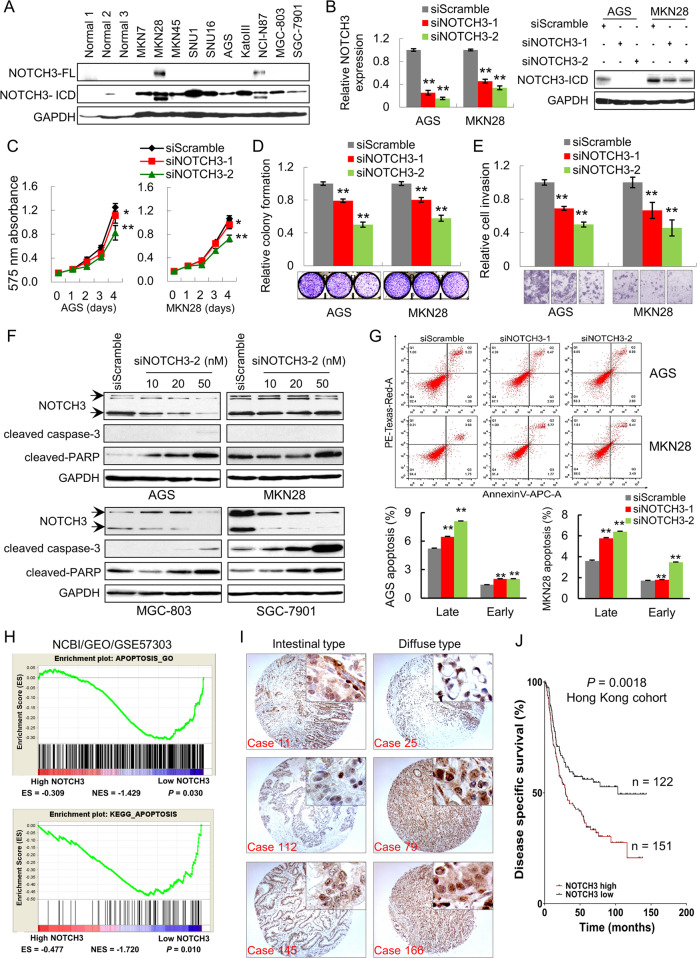
NOTCH3 knockdown exerts anti-oncogenic effects in vitro. **A** NOTCH3 is overexpressed in all ten GC cell lines compared with normal gastric tissues. **B** The mRNA and protein expression of NOTCH3 after siRNA-mediated knockdown in AGS and MKN28 cells. **C** NOTCH3 knockdown by two siRNAs suppressed AGS and MKN28 cell proliferation (*P < 0.05; **P < 0.001). **D** NOTCH3 silencing decreased monolayer colony formation ability of the cancer cells (**P < 0.001). **E** NOTCH3 deletion inhibited cell invasion ability of GC cells (**P < 0.001). **F** Western blot analysis demonstrated the activation of cleaved caspase-3 and cleaved-PARP induced by NOTCH3 knockdown in all four GC cells. **G** Flow cytometry for apoptosis analysis of siScramble and siNOTCH3 transfectants. The representative bar chart of cell distribution was selected from two independent experiments with the same results (**P < 0.001). **H** Enrichment plots of apoptotic gene expression signatures according to NOTCH3 expression in NCBI/GEO/GSE57303 cohort (upper panel, APOPTOSIS_GO, *P* = 0.039; lower panel, KEGG_APOPTOSIS, *P* = 0.010). The barcode plot indicated the position of the genes in each gene set; red and blue colors represented the high and low expression of NOTCH3, respectively. ES enrichment score, NES normalized enrichment score. **I** The immunohistochemistry images of NOTCH3 in primary GC samples. NOTCH3 was detected both in the cytoplasm and nuclei of the intestinal and diffuse-type cancer cells. **J** NOTCH3 nuclear accumulation was associated with poor disease-specific survival (*P* = 0.0018).

NOTCH3 expression was examined in GC cell lines and normal gastric epithelium tissue by western blot. NOTCH3 was upregulated in all ten GC cell lines compared with the normal controls (Fig. 2A). To investigate the biological function of NOTCH3 in GC, we performed siRNA-mediated NOTCH3 knockdown in GC cell lines. siNOTCH3 downregulated its mRNA and protein expression in AGS and MKN28 cells (Fig. 2B). The expression correlation between NOTCH3 and other family members were all positive (TCGA cohort) (Supplementary Fig. S1A). However, knocking down NOTCH3 did not decrease the expression of NOTCH1 and NOTCH4, only a 30–40% reduction of NOTCH2 expression was observed (Supplementary Fig. S1B). NOTCH3 silencing inhibited cell proliferation in the two GC cell lines (Fig. 2C). Consistently, NOTCH3 knockdown suppressed monolayer colony formation ability of these cells (Fig. 2D). Cell invasion ability was also impaired after NOTCH3 knockdown in AGS and MKN28 cells (Fig. 2E). To elucidate the mechanisms of NOTCH3 depletion in impeding cell growth, apoptosis makers were examined. Cleaved caspase-3 and cleaved-PARP, serving as apoptosis markers, were activated in a concentration-dependent manner (Fig. 2F). Flow cytometry assay confirmed that siNOTCH3 induced apoptosis, as both AGS and MKN28 cells exhibited accumulative apoptotic cell ratio in the siNOTCH3 cells (Fig. 2G). Moreover, gene set enrichment analysis using NCBI/GEO/GSE57303 dataset demonstrated that cell apoptosis-related genes were significantly enriched in cases with low NOTCH3 expression (Fig. 2H) [15]. NOTCH3 expression was detected in the cytoplasm and nucleus of the cancer cells in both intestinal and diffuse-type GC samples (Fig. 2I). The variant expressional patterns of NOTCH3 might be due to the fact that when the receptor is activated, NICD3 is translocated into the nucleus. NOTCH3 nuclear accumulation was associated with worse disease-specific survival in Hong Kong tissue microarray cohort consisting of 273 primary GC samples (Fig. 2J). The correlation of NOTCH3 nuclear accumulation with the other clinicopathological parameters was summarized in Supplementary Table S1. There was no correlation between NOTCH3 expression and sex, age, cancer type, grade, TNM stage, lymph node metastasis, or *H. pylori* infection. By univariate and multivariate Cox regression analysis, NOTCH3 expression, elder age, and advanced TNM stage were independently associated with disease-specific survival (Supplementary Table S2).

### NOTCH3 is negatively regulated by miR-491-5p and miR-875-5p

**Fig. 3:**
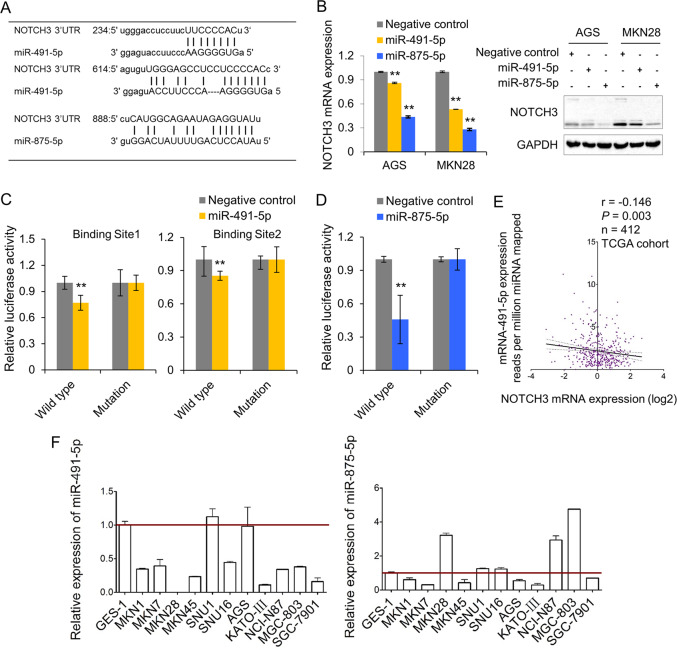
NOTCH3 is negatively regulated by miR-491-5p and miR-875-5p. **A** Predicted putative miR-491-5p and miR-875-5p binding sites in 3′UTR of NOTCH3. **B** Both mRNA and protein expression of NOTCH3 after miR-491-5p and miR-875-5p overexpression in AGS and MKN28 cells (**P < 0.001). **C** miR-491-5p inhibited the luciferase activity of constructs encompassing the wild-type binding sites in NOTCH3 3′UTR (**P < 0.001). **D** The relative luciferase activity of constructs containing the binding site in NOTCH3 3′UTR was quenched by miR-875-5p (**P < 0.001). **E** miR-491-5p was negatively correlated with NOTCH3 mRNA expression in TCGA cohort (*r* = −0.146, *P* = 0.003). **F** The expression of miR-491-5p and miR-875-5p in GC cells compared with an immortalized gastric epithelium cell GES-1.

From the TCGA dataset, copy number aberrations did not correlate with NOTCH3 mRNA expression. We thus hypothesized that post-transcriptional regulation might play an important role in regulating NOTCH3 expression. As predicted by microRNA.org (www.microRNA.org), 12 miRNAs putatively targeting NOTCH3 with best mirSVR scores were listed in Supplementary Table S3. Most of these miRNAs were also predicted by TargetScan (www.targetscan.org). miR-875-5p and miR-491-5p were listed as the top hits among the 12 miRNAs. In the 3′UTR of NOTCH3, there were two binding sites for miR-491-5p and one binding site for miR-875-5p (Fig. 3A). Ectopic expression of miR-875-5p and miR-491-5p decreased NOTCH3 mRNA and protein expression (Fig. 3B). To test whether miR-491-5p or miR-875-5p directly binds to the 3′UTR of NOTCH3, luciferase activity assays were conducted. miR-491-5p or miR-875-5p overexpression inhibited the luciferase activities of the reporters containing wild-type binding sites, but in the constructs containing mutant binding sites, the inhibitory effect was abolished (Fig. 3C, D). Furthermore, NOTCH3 mRNA expression was negatively correlated with miR-491-5p expression in TCGA cohort (Fig. 3E), suggesting a regulatory effect of this tumor suppressor miRNA on NOTCH3 in GC. The expression of miR-491-5p and miR-875-5p in GC cell lines were next examined. miR-491-5p and miR-875-5p were downregulated in nine and six GC cell lines, respectively, compared with GES-1, an immortalized normal gastric epithelium cell line (Fig. 3F).

### miR-491-5p and miR-875-5p are tumor-suppressive miRNAs

**Fig. 4:**
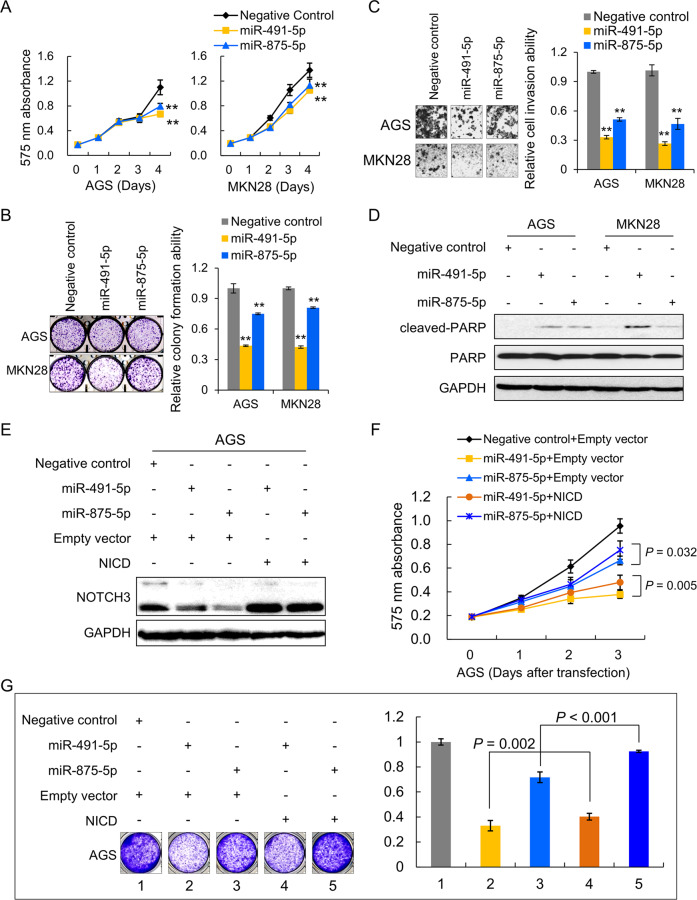
miR-491-5p and miR-875-5p exert antitumor effect and NOTCH3 re-expression partly rescues the suppressive effect of miR-491-5p and miR-875-5p. **A** MTT proliferation assays of AGS and MKN28 cells with ectopic miR-491-5p and miR-875-5p expression (**P < 0.001). **B** Monolayer colony formation ability was suppressed by ectopic expression of miR-491-5p and miR-875-5p in cancer cells (**P < 0.001). **C** Overexpressed miR-491-5p and miR-875-5p significantly inhibited cell invasion in AGS and MKN28 (**P < 0.001). **D** Cell apoptosis marker, cleaved-PARP, was activated after ectopic expression of miR-491-5p and miR-875-5p. **E** Re-expression of NOTCH3 was confirmed by western blot in the rescue experiments. **F** Cell proliferation ability was partly restored in miR-491-5p or miR-875-5p transfectants after re-expression of NOTCH3. **G** Re-expression of NOTCH3 partly diminished the tumor-suppressive effect of miR-491-5p and miR-875-5p in monolayer colony formation assays.

The functional role of miR-491-5p or miR-875-5p was tested in AGS and MKN28 cells. Ectopic expression of miR-491-5p and miR-875-5p significantly suppressed cell proliferation (Fig. 4A) and inhibited the monolayer colony formation ability (Fig. 4B). Overexpression of these two miRNAs could also quench cell invasion ability (Fig. 4C). miR-491-5p or miR-875-5p induced apoptosis and activated cleaved-PARP expression, concordant with the phenotype changes of NOTCH3 knockdown (Fig. 4D). To test if NOTCH3 is the main target of miR-491-5p and miR-875-5p, rescue experiments were performed. Ectopic expression of NOTCH3 in the AGS cells expressing miR-491-5p and miR-875-5p cells (Fig. 4E) partly rescued the suppressed cell proliferation induced by these miRNAs (Fig. 4F). Moreover, NOTCH3 NICD re-expression reversed the inhibitory effect of miR-491-5p and miR-875-5p on monolayer colony formation (Fig. 4G). Taken together, NOTCH3 is a functional target negatively regulated by tumor-suppressive miR-491-5p and miR-875-5p.

### Identification of PHLDB2 as the key downstream effector of NOTCH3

**Fig. 5:**
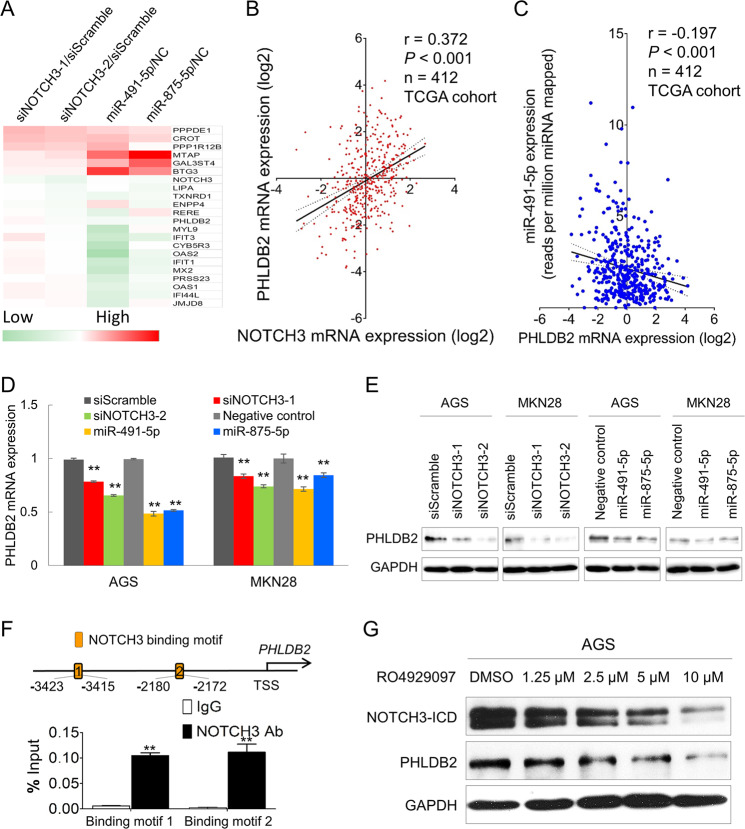
PHLDB2 is the key downstream modulator of miR-491-5p/miR-875-5p-NOTCH3 cascade in GC. **A** The top-listed genes which were regulated by NOTCH3 knockdown or miR-491-5p/miR-875-5p overexpression. **B** PHLDB2 is positively correlated with NOTCH3 mRNA expression in TCGA primary GC tissues (*r* = 0.372, *P* < 0.001). **C** PHLDB2 mRNA expression is negatively associated with miR-491-5p (*r* = −0.197, *P* < 0.001). **D** PHLDB2 mRNA was downregulated after NOTCH3 knockdown or miR-491-5p/miR-875-5p overexpression in AGS and MKN28 cells (**P < 0.001). **E** Both NOTCH3 knockdown or miR-491-5p/miR-875-5p overexpression decreased the protein expression of PHLDB2 by western blot analysis. **F** ChIP-qPCR experiments revealed that NOTCH3 directly interacts with the two NOTCH3-CSL binding motifs in AGS cells (**P < 0.001). **G** Expression of NOTCH3-ICD and PHLDB2 after treating AGS cells with RO4929097.

Given that siNOTCH3 and miR-491-5p/miR-875-5p induced apoptosis, but had no effect on cell cycle arrest and the expression of cell cycle regulators such as CCND1, CDK4, CDK6, p21, p27, and p-Rb (Supplementary Fig. S1C). We first evaluated the expression correlation of NOTCH3 with epithelial–mesenchymal transition (EMT) and cancer stemness markers. The expression correlation of NOTCH3 and three main EMT markers (CDH1, CDH2, VIM) and a stemness marker (ALDH1A1) was checked by using TCGA dataset. NOTCH3 has positive correlation with Vimentin (VIM) and N-Cadherin (CDH2), but conversely correlated with E-Cadherin (CDH1), suggesting NOTCH3 might be involved in the EMT. However, for the stemness marker correlation analysis, NOTCH3 did not exhibit co-expression with ALDH1A1 together with other stemness markers, suggesting NOTCH3 has little impact on keeping the cancer stemness in gastric tumorigenesis (Supplementary Fig. S1D). We then sought to identify downstream factors in mediating the antiapoptotic and pro-metastasis effect of NOTCH. We initially examined the expression of known NOTCH3 downstream factors, such as HEY1, HES1, OCT4, SOX2, and NANOG. However, no significant changes in the expression of these genes were found after knockdown NOTCH3 (Supplementary Fig. S1E). Thus, expression profiling was employed to comprehensively unravel differentially expressed genes after NOTCH3 knockdown or miR-491-5p/miR-875-5p overexpression (Supplementary Table S4). The top-ranked up- and down-regulated genes in both NOTCH3 knockdown and miR-491-5p/miR-875-5p overexpression transfectants were shown in Fig. 5A. We then evaluated the expression correlation of several top candidate genes with NOTCH3 in TCGA cohort and confirmed PHLDB2 is the only candidate which shows a significant positive correlation with NOTCH3 in human GC (Fig. 5B). While the other candidates such as LIPA, ENPP4, and TXNRD1 failed to positively correlate with NOTCH3 expression (Supplementary Fig. S1F). In addition, PHLDB2 expression was found to be negatively correlated with miR-491-5p expression in primary samples (Fig. 5C). Either NOTCH3 knockdown or miR-875-5p/miR-491-5p overexpression decreased PHLDB2 expression at both mRNA (Fig. 5D) and protein levels (Fig. 5E). Bioinformatic analysis identified two NOTCH3 binding motifs in the promoter region of *PHLDB2*. Through ChIP-qPCR assay, these two binding motifs were confirmed to directly interact with NOTCH3 (Fig. 5F). To further confirm the binding affinity in the regulatory process, AGS cells were treated with GSI (RO4929097). Both cleaved NOTCH3-ICD and PHLDB2 were decreased in a RO4929097 dose-dependent manner (Fig. 5G), suggesting NOTCH3 was required for PHLDB2 expression.

### PHLDB2 plays an oncogenic role in gastric carcinogenesis

**Fig. 6:**
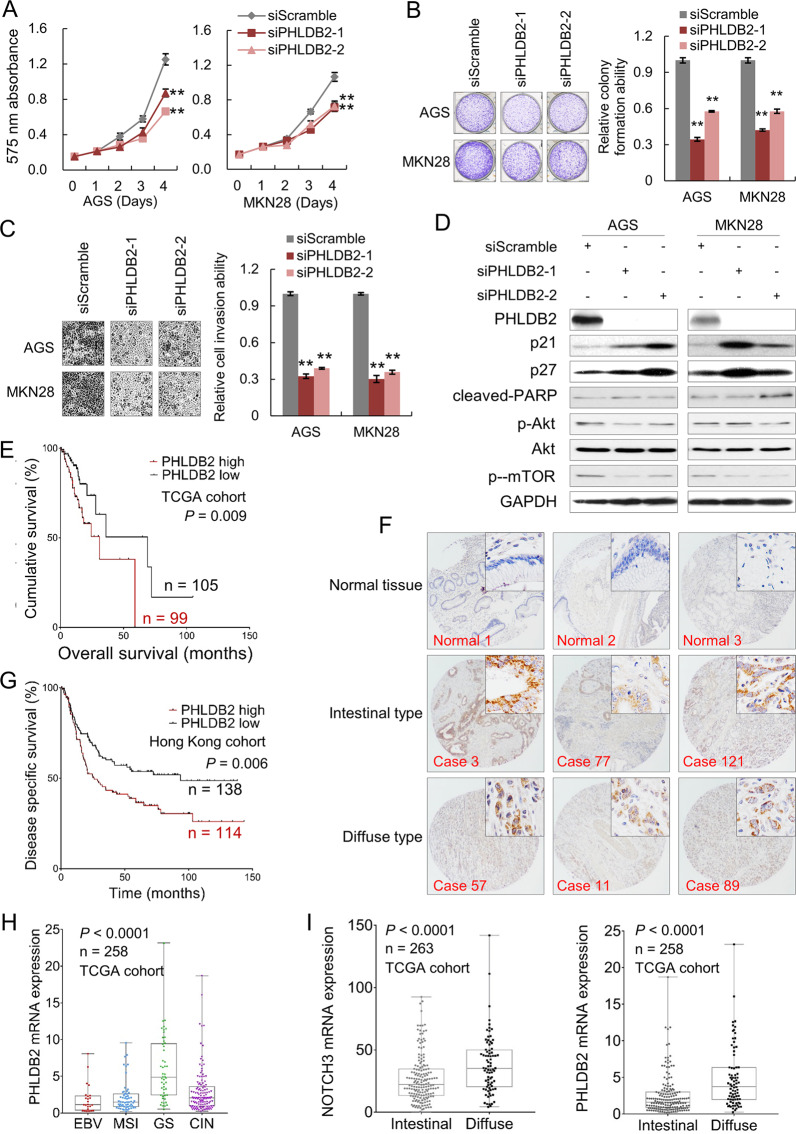
PHLDB2 plays an oncogenic role and its overexpression correlates with poor survival in GC. **A** siPHLDB2 inhibited cell proliferation in AGS and MKN28 cell lines (**P < 0.001). **B** PHLDB2 silencing decreased monolayer colony formation ability (**P < 0.001). **C** Tumor cell invasion ability was suppressed by PHLDB2 knockdown (**P < 0.001). **D** Western blot analysis demonstrated elevated expression of cell cycle regulator proteins, p21 and p27, together with activated apoptosis marker cleaved-PARP after silencing PHLDB2 and inactivated Akt and mTOR in GC cells. **E** Upregulation of PHLDB2 is correlated with poor overall survival in GC patients (TCGA cohort, *P* = 0.009). **F** Immunohistochemistry images of PHLDB2 in primary GC samples. PHLDB2 showed negative expression in normal epithelium cells, but it was expressed in the membrane and cytoplasm of the cancer cells. **G** PHLDB2 abundance was associated with poor disease-specific survival in Hong Kong cohort (*P* = 0.006). **H** The expression of *P*HLDB2 in the four molecular subtypes of GC. **I** NOTCH3 and PHLDB2 were overexpressed in diffuse-type GC compared with intestinal type GC (TCGA cohort; NOTCH3, *P* < 0.001; PHLDB2, *P* < 0.001).

PHLDB2, short for Pleckstrin Homology-Like Domain Family B Member 2, has been reported to regulate cell migration, adhesion, and invasion of colon cancer cell lines [16, 17]. AGS and MKN28 cells were used to investigate the functional role of PHLDB2. Knocking down PHLDB2 inhibited cell proliferation (Fig. 6A), suppressed colony formation (Fig. 6B), and significantly decreased cell invasion ability in these two cell lines (Fig. 6C). Its depletion activated the protein expression of p21, p27, and cleaved-PARP. As previously reported, PHLDB1 enhances the activation of Akt-mTOR signaling [18] and it is a crucial mechanism for cell viability. We then investigated whether PHLDB2 regulates Akt signaling. As observed, PHLDB2 knockdown suppressed the activation of Akt and mTOR (Fig. 6D). PHLDB2 upregulation was associated with poor overall survival in TCGA cohort (Fig. 6E). By immunohistochemistry (IHC), PHLDB2 was found to be predominantly located in the cytoplasm of the cancer cells in both intestinal and diffuse-type GC of Hong Kong cohort (Fig. 6F). GC patients with high PHLDB2 expression showed worse survival, which was consistent with the TCGA cohort (Fig. 6G). Clinical correlation analysis demonstrated that cytoplasmic PHLDB2 abundance was associated with elder age (*P* = 0.011, Supplementary Table S5). By univariate Cox regression analysis, elder age, diffuse type, high grade, advanced T, N, and M stage, lymph node metastasis and high expression of PHLDB2 were associated with poor outcome. However, by multivariate Cox analysis, PHLDB2 abundance cannot independently indicate poor outcome (*P* = 0.194, Supplementary Table S6). Among the four TCGA molecular subtypes of GC, PHLDB2 was highly expressed in the GS subtype, consistent with the pattern of NOTCH3 expression (Fig. 6H). In the TCGA dataset, diffuse-type GC demonstrated high NOTCH3 and PHLDB2 mRNA expression, indicating that NOTCH3 and PHLDB2 were involved in promoting metastasis of GC cells (Fig. 6I).

### Subgrouping primary samples according to NOTCH3-PHLDB2-Akt expression

**Fig. 7:**
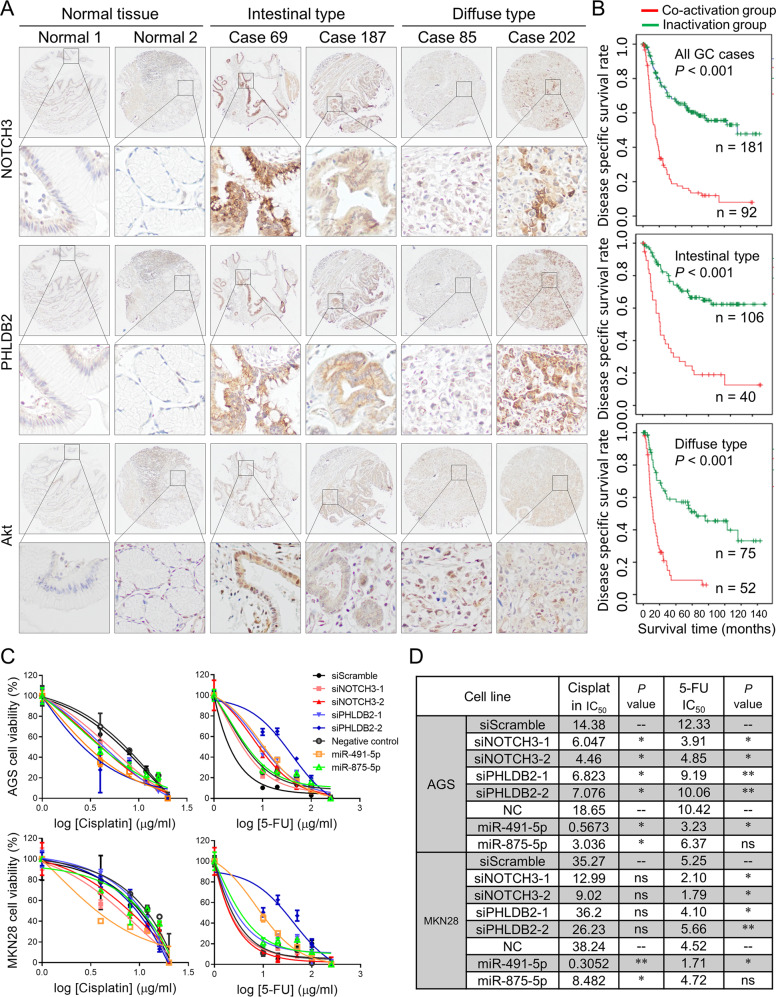
Stratifying primary GC samples as NOTCH3-PHLDB2-Akt co-activation and inactivation subgroups. **A** Representative images of immunohistochemistry staining for NOTCH3, PHLDB2, and Akt in primary GC samples. **B** NOTCH3-PHLDB2-Akt co-activation group was associated with poor outcomes both in intestinal and diffuse-type GC. **C** AGS and MKN28 cells with NOTCH3/PHLDB2 knockdown or miR-491-5p/miR-875-5p overexpression were more sensitive to Cisplatin or 5-FU treatment, compared with siScramble or Negative control, respectively (*P* < 0.05). **D** The summarized IC_50_ of Cisplatin and 5-FU on AGS and MKN28 cells with NOTCH3/PHLDB2 knockdown or miR-491-5p/miR-875-5p overexpression (*P < 0.05; **P < 0.001).

To investigate the therapeutic implication of the miR-491-5p/miR-875-5p-NOTCH3-PHLDB2 cascade in gastric carcinogenesis, we stratified a group of patients with co-positive NOTCH3, PHLDB2, and Akt by tissue microarray. Through IHC analysis, these factors showed very weak signal in normal tissues. Co-activation of these factors were identical in 33.7% (92 out of 273) GC cases. Particularly, 27.4% of co-activation cases were found in intestinal type GC and 40.9% of cases represented (52 out of 127) in diffuse-type GC (Fig. 7A). According to the IHC result, clinical association of the NOTCH3-PHLDB2-Akt co-activation group were analyzed. As indicated in Fig. 7B, the disease-specific survival rate was significantly reduced in co-activation group compared with inactivation group. Thus, NOTCH3-PHLDB2-Akt was considered as a promising therapeutic target. To further test the therapeutic potential of this axis in gastric carcinogenesis, we treated AGS and MKN28 cells, which subjected to NOTCH3/PHLDB2 depletion or miR-491-5p/miR-875-5p overexpression in prior, with chemotherapeutic agents Cisplatin or 5-FU. As shown in Fig. 7C, cells with NOTCH3/PHLDB2 depletion or miR-491-5p/miR-875-5p were more sensitive to Cisplatin or 5-FU, as determined by MTT cell proliferation assays. Corresponding IC_50_ values of these AGS and MKN28 transfectants were listed (Fig. 7D).

### Targeting the oncogenic NOTCH3-PHLDB2-Akt axis by small molecules

**Fig. 8:**
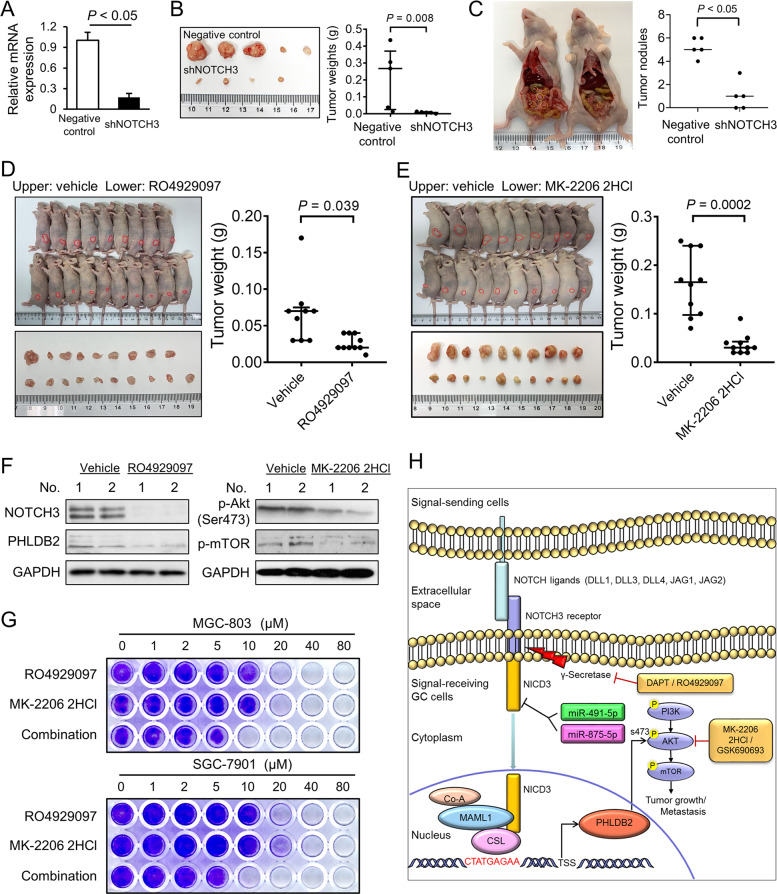
Targeting NOTCH3-PHLDB2-Akt signaling cascades by small molecule inhibitors in GC. **A** Knockdown efficiency of shNOTCH3 was validated in MGC-803 cells (*P* < 0.05). **B** Knockdown of NOTCH3 by shRNA significantly suppressed the xenograft formation in nude mice compare with negative control (*n* = 5, *P* = 0.008). **C** The shNOTCH3 group formed smaller and less number of nodules than the negative control group (*P* < 0.005). **D** RO4929097 inhibited subcutaneous tumor growth in nude mice (Vehicle group *n* = 9, RO4929097 group *n* = 10, *P* = 0.039). **E** MK-2206 2HCl suppressed xenograft formation of the MGC-803 cells (*n* = 10, *P* = 0.0002). **F** Western blot analysis: NOTCH3-ICD and PHLDB2 expression after RO4929097 treatment; Akt-mTOR phosphorylation upon MK-2206 2HCl. Two tumor samples were applied for detecting the related protein levels. **G** Monolayer colony formation images of MGC-803 and SGC-7901 cells treated with RO4929097 or MK-2206 2HCl alone, or a combination of these two inhibitors. **H** Overall schematic presentation of miR-491-5p/miR-875-5p-NOTCH3-PHLDB2-Akt cascade in gastric carcinogenesis.

To evaluate the therapeutic potential of targeting NOTCH3 in GC, an shRNA commercially available against NOTCH3 was employed to decrease the expression of NOTCH3 in MGC-803. Knockdown efficiency was validated by qRT-PCR (Fig. 8A). The shNOTCH3 transfectants were inoculated subcutaneously into the nude mice. shNOTCH3 significantly suppressed growth of xenografts compared with the negative control (Fig. 8B). Then the xenografts were cut into small pieces and transplanted into the peritoneum cavity of the nude mice with diluted Matrigel. The shNOTCH3 groups formed smaller and less number of nodules than the negative control group, suggesting NOTCH3 knockdown inhibits the peritoneal metastatic abilities of the cancer cells (Fig. 8C). To test the efficacy of GSI in vivo, we treated MGC-803 xenografts with GSI inhibitor RO4929097. RO4929097 significantly quenched xenograft growth compared with PBS vehicle control (Fig. 8D). Since silencing PHLDB2 caused inactivation of Akt signaling, an Akt inhibitor MK-2206 2HCl was administrated in xenograft formation. MK-2206 2HCl significantly retarded the growth of xenografts (Fig. 8E). By western blot, the expression of NOTCH3-ICD and PHLDB2 were confirmed to be suppressed in the xenografts treated with RO4929097. The efficacy of MK-2206 2HCl was also validated based on the decreased phosphorylation of Akt-mTOR (Fig. 8F). Monolayer colony formation abilities of MGC-803 and SGC-7901 were impaired by either single small molecule treatment or combinatorial administration in a dose-dependent manner (Fig. 8G). To summarize, the activation of NOTCH3 in gastric carcinogenesis is partly due to the silence of tumor suppressor miRNAs, miR-491-5p, and miR-875-5p. As a transcription co-activator, NOTCH3 exerts its oncogenic role directly through PHLDB2. The miR-491-5p/miR-875-5p-NOTCH3-PHLDB2 regulatory cascade is involved in gastric carcinogenesis and promotes cancer progression (Fig. 8H).

## Discussion

Notch signaling is an evolutionarily conserved pathway important for the cell-fate determination in the developing embryo and mature tissues. Stem cell maintenance, binary cell-fate decisions, and induction of differentiation are the three main functions of Notch signaling in self-renewing tissues [19]. Mammals have four Notch receptors (NOTCH1-4) and five ligands, Delta-like ligand 1 (DLL1), Delta-like ligand 3 (DLL3), Delta-like ligand 4 (DLL4), Jagged1 (JAG1), and Jagged2 (JAG2) [20]. Some genes are consistently upregulated by activated Notch across multiple tissue types, such as hairy and enhancer of split, hairy/enhancer-of-split related with YRPW motif families, CCND1, NF-κB, NANOG, and c-Myc [21–25].

The functional role of Notch has also been identified in carcinogenesis. Depending on cancer types, Notch signaling has mostly been associated with oncogenic and growth-promoting roles. In colorectal, breast and pancreas cancers [26–28], Notch signaling is a crucial oncogenic driver. In colorectal cancer (CRC), NOTCH3 transcript is significantly upregulated in primary and metastatic samples, and it accelerates tumor growth [29]. Nuclear NOTCH3 accumulation is associated with tumor recurrence in stage II and III CRC patients [30]. NOTCH3 directly upregulates the expression of MUSASHI-1 (MSI-1), a well-established stem cell marker in both normal and malignant colon epithelium cells [31]. On the contrary, Notch signaling may serve as a tumor suppressor in other malignancies such as in skin [32] and lung cancer [33]. Thus, the functional roles of Notch in solid tumors seem highly context dependent.

In GC, most of the research focuses on NOTCH1. NOTCH1 activation is a poor prognostic factor in GC patients [34]. Increased NOTCH1 is associated with tumor differentiation, depth of tumor invasion, lymph node metastasis, and Lauren classification. NOTCH1 activation is partly due to the epigenetic regulation of the Notch ligand DLL1 [35], and its activation promotes GC through upregulation of COX-2 [8]. Overexpression of NOTCH1 also promotes the interaction between nuclear STAT3 and Twist promoter region, thereby activating the NOTCH1/STAT3/TWIST signaling axis [36]. On the other hand, the roles of NOTCH2/3/4 in GC are largely unexplored. High NOTCH2 expression is correlated with poor survival and it serves as a prognostic marker in GC [37]. miR-23b was reported to interplay with NOTCH2 to form a reciprocal regulation loop [38]. However, NOTCH2 was also reported to function as a tumor suppressor by inhibiting the PI3K/AKT pathway in MKN45 cells [39]. NOTCH3 overexpression is associated with the intestinal type GC and better histological differentiation, suggesting that it might be a favorable prognostic indicator [40]. NOTCH4 induces the expression and activation of Wnt/β-catenin to regulate GC growth [41]. Here, we revealed that NOTCH3 is uniformly upregulated in GC and its expression is correlated with worse outcome from multiple datasets, supporting the importance of NOTCH3 as a druggable target in GC. Moreover, NOTCH3 depletion enhances the cellular sensitivity to the anticancer drugs, providing a rational for a novel strategy combining NOTCH3 inhibitors with chemotherapy for NOTCH3-postive GC treatment.

Transcriptional or post-transcriptional regulation might underlie NOTCH3 activation in GC. We provided the first evidence that NOTCH3 is negatively regulated by tumor-suppressive miRNAs, miR-491-5p, and miR-875-5p. These two miRNAs were initially discovered as tumor suppressors in CRC. miR-491-5p was reported to exert tumor-suppressive function in breast, ovarian, cervical, and non-small cell lung cancers [42–44]. miR-875-5p promoted apoptosis in colon cancer cells by upregulation of key apoptosis protein cleaved caspase-3 [45]. Our work also identified PHLDB2 as a novel and potent downstream effector of NOTCH3 in promoting gastric carcinogenesis. PHLDB2 is positively correlated with NOTCH3, but it shows negative association with miR-491-5p. In agreement with our findings, the upregulation of PHLDB2 was associated with poor overall survival in CRC patients. PHLDB2 has also been reported as a direct target of miR-29c-3p, a tumor-suppressive miRNA inhibiting migration and invasion of CRC cells [17].

Small molecules or compounds targeting NOTCH3 signaling have been used for clinical trials. Several classes of Notch inhibitors have been developed, including GSIs, siNotch delivery systems, and monoclonal antibodies against Notch receptors or ligands [46]. GSIs are small molecules that inhibit Notch cleavage and they might be applicable in suppressing cancer cell proliferation in conjunction with chemotherapy [47]. In this study, we employed RO4929097 to inhibit NOTCH3 both in vitro and in vivo, and found that NOTCH3-ICD and PHLDB2 expression were abrogated after treatment, suggesting GC patients with NOTCH3-PHLDB2 activation might benefit from GSI. RO4929097 was previously evaluated in some phase I and phase II clinical studies of advanced solid tumors, such as pancreatic cancer, melanoma, and metastatic CRC [48–51]. However, RO4929097 was not beneficial to suppress tumor growth or promote apoptosis [52]. Actually, the therapeutic potential of Notch inhibitors is not well-supported by the current preclinical and clinical trials in GC. In this study, we revealed Notch crosstalks Akt signaling. Akt proteins are serine-threonine kinases activated by PI3K to accelerate cell survival and inhibit apoptosis [53, 54]. Its activation and overproduction are strongly associated with chemo or radioresistance [55]. MK-2206 is an active allosteric Akt inhibitor effective for multiple types of solid tumors. It is highly sensitive and selective to Akt enzymes through binding on the Akt proteins. Combinational administration of MK-2206 with first-line anticancer drugs was found to produce synergistic effects on suppressing tumor growth [56]. MK-2206 was employed with lapatinib, an EGFR/human HER2/neu dual inhibitor, on GC cells with *HER2* amplification [56]. RAD001 and MK-2206 co-administration promotes ERK-dependent autophagic cell death in *PTEN* Mutant GC cells [57]. In our study, we evaluated the synergistic effect of co-targeting Akt and NOTCH3 in GC cells. The combinational usage of these two inhibitors significantly enhanced the cell death in a higher efficacy than any single drug administration, which provided a novel therapeutic strategy for NOTCH3-postive GCs.

## Conclusions

In conclusion, our study revealed a druggable miR-491-5p/miR-875-5p-NOTCH3-PHLDB2 cascade in gastric carcinogenesis. NOTCH3, as the central part of the cascade, promotes tumor progression through inhibition of apoptosis. Its activation in GC is partly due to the silence of tumor-suppressive miRNAs, miR-491-5p, and miR-875-5p. PHLDB2 is a crucial downstream effector of NOTCH3 and transduces its oncogenic effect to Akt signaling. Our study identified multiple prognostic biomarkers of GC and demonstrated the therapeutic potential of NOTCH3 inhibitors in combination with traditional chemotherapy for GC treatment.

## Materials and methods

### Cell cultures and clinical samples

MKN1, MKN7, MKN28, MKN45, SNU1, SNU16, AGS, KatoIII, NCI-N87, MGC-803, SGC-7901, TMK-1, and GES-1 cells were cultured as reported [58]. A total of 273 primary GC samples were retrieved in Prince of Wales Hospital of The Chinese University of Hong Kong (CUHK) between 1996 and 2006. The formalin-fixed paraffin-embedded blocks of these cases were used for the study. According to protocols approved by CUHK Clinical Research Ethics Committee, written informed consent of the human samples was obtained from patients. The CUHK Clinical Research Ethics Committee approved the usage of these human samples and the Reference No. is 2017.091.

### Protein extraction and western blot analysis

Western blot was performed with whole-cell protein lysates in RIPA lysis buffer. Equal amounts of 10 µg proteins were used for western blot analyses. PHLDB2 primary antibody was from Abcam (1:1000, ab202350, Cambridge, UK). Other primary antibodies were from Cell Signaling (Danvers, MA, US), including NOTCH3 (D11B8) (1:1000, #5276), p21 (1:1000, #2946), p27 (1:1000, #2552), Phospho-Rb (Ser807/811) (1:1000, #9308), cleaved caspase-3 (Asp175) (1:1000, #9661), cleaved-PARP (Asp214) (1:1000, #9541), Phospho-Akt (Ser473) (1:1000, #4060), Phospho-mTOR (Ser2448) (1:1000, #5536), CCND1 (1:1000, #2978), CDK4 (1:1000, #12790), CDK6 (1:1000, #3136), and GAPDH (1:2000, #2118). Secondary antibodies anti-Mouse IgG-HRP (1:30000, 00049039) and anti-Rabbit IgG-HRP (1:10000, 00028856) were from Dako (Glostrup, Denmark).

### IHC staining

NOTCH3 and PHLDB2 IHC were performed on the tissue microarray containing 273 GC cases. NOTCH3 primary antibody (1:100, #5276) was from Cell Signaling. The nuclear accumulation of NOTCH3 was assessed according to the ratio of GC cells with positive nuclear staining (low expression, ≤10%; high expression, >10%). PHLDB2 antibody was from Abcam (1:100, ab202350). Akt antibody was from Cell Signaling (1:100, CST#9272). The cytoplasmic staining of PHLDB2 was quantified according to the proportion of positive tumor cells and the intensity of cytoplasmic staining.

### RNA extraction and qRT-PCR analyses

Total RNA was extracted using RNAiso Plus (Takara, Japan) according to the manufacturer’s instructions. RNA concentrations were measured by NanoDrop ND-2000 instrument (USA). Complementary DNA (cDNA) was synthesized with High-Capacity cDNA Reverse Transcription Kit (Applied Biosystems, Carlsbad, CA). qRT-PCR was used to examine the mRNA expression in GC cell lines. The primers of qRT-PCR used in this study were listed in Supplementary Table S7.

### miRNA and siRNA transfection for functional studies

miR-491-5p (PM11479) and miR-875-5p (PM12450) precursors, and scramble control (AM17110), were from Life Technologies (Carlsbad, CA). siRNAs, including siNOTCH3-1 (SI00009499), siNOTCH3-2 (SI00009513), siPHLDB2-1(SI00684040), siPHLDB2-2 (SI04190116), and Scramble siRNAs (SI03650318) were from Qiagen (Valencia, CA). Lipofectamine 2000 (Invitrogen) was employed for all transfection assays. Functional assays (MTT proliferation, monolayer colony formation, and cell invasion) were described previously [59]. All flow cytometry for the apoptosis and cell cycle distribution analysis were performed with BD FACSCalibur (BD Biosciences Company, USA). The apoptotic cells were detected with Annexin V-APC apoptosis detection kit as described [58].

### Luciferase reporter assay

The putative miR-491-5p and miR-875-5p binding sites in 3′UTR of NOTCH3, as well as the mutant binding sites were sub-cloned into pMIR-REPORT vector (Ambion, Grand Island, NY). The sense and antisense of oligonucleotides were listed in Supplementary Table S8. MKN28 cells were co-transfected with luciferase reporter constructs containing either wild-type sequence or the mutant counterpart, and pRL-TK Renilla reporter for 36 h. Cells were lysed for luciferase activity test by dual-luciferase reporter assay system (Promega, Madison, WI).

### Expression microarray profiling

Total RNAs were extracted from siScramble, siNOTCH3-1, siNOTCH3-2, Negative control, miR-491-5p, and miR-875-5p transfected AGS cells using RNeasy kit (Qiagen, Valencia, CA). All samples were analyzed by the Humanizing Genomics Macrogene Company (Seoul, Republic of Korea) according to mRNA expressional microarray protocols.

### Chromatin immunoprecipitation and qPCR

Chromatin immunoprecipitation (ChIP) assays were performed using NOTCH3 antibody (#5276, Cell Signaling). In brief, AGS cells were seeded in 15 cm culture dish and crosslinked with 1% formaldehyde for 10 min. Cells were pelleted and sonicated for shearing DNA to fragments between 200 and 500 bps. Sonicated chromatin was centrifuged and then incubated with 1 µg NOTCH3 antibody or normal IgG for overnight. The precipitated DNA was analyzed by qPCR. Primers were used as follows: binding motif 1 (F: GGC AGT CCT AGG TTT GCA AT; R: TCA AAC CCA GTT CTA GGC AAA A); binding motif 2 (F: GTA GAG GAC CCA TAA CTT GCA; R: TGC TAG CCT AAA TTA GTC AGC C).

### Drug sensitivity test

Cisplatin and 5-FU were commercially available from Sigma-Aldrich (St. Louis, MO, USA). The half maximal inhibitory concentration (IC_50_) was evaluated before drug sensitivity test. The transfected cells were seeded in 96-well plated and treated with anticancer drugs for 24 h. The cytotoxicity of Cisplatin and 5-FU in GC cell lines (AGS and MKN28) was measured by MTT proliferation assays.

### Animal model

To check the tumor growth and peritoneal metastasis inhibition effect after NOTCH3 depletion, the NOTCH3 was downregulated by shRNA-mediated knockdown in MGC-803 cells (shNOTCH3 plasmid, sc-37135-SH, Santa Cruz). Then the shNOTCH3 transfected cells and their counterparts were subcutaneously injected into the dorsal flank of Balb/c nude mice (4 to 6-week old). Three weeks later, the xenografts were dissected and measured. The xenografts were then cut into small pieces (1.0–2.0 mm^3^) and inoculated into the peritoneum of the nude mice for another 6 weeks to investigate the metastasized tumor growth in the peritoneum. For the small molecule tests, the MGC-803 cells were subcutaneously injected into the nude mice. Notch GSI (RO4929097, Selleckchem, Houston, TX) and Akt inhibitor MK-2206 2HCl were intraperitoneally injected every 4 days for five times with 10 mg/kg dosages. Body and tumor weights were measured after 21-day inoculation. The animal handling and experimental procedures were approved by Department of Health, Hong Kong (Reference No: 16-561 in DH/HA&P/8/2/1 Pt.61).

### Statistical analysis

Associations between NOTCH3 or PHLDB2 expression and clinicopathological parameters were achieved by nonparametric analysis. Kaplan–Meier curves were fitted to overall and disease-free survival data. Statistical difference of two groups was calculated by two-tailed Student’s *t*-test and correlation of expression was analyzed by Spearman correlation test. Drug responses between multiple groups were compared by two-way ANOVA with post-hoc comparison of each two groups. Differences were considered significant when *P* < 0.05. Analyses were performed using GraphPad Prism software (Intuitive Software for Science) and SPSS 17.0 statistical software (SPSS Inc.).

## Supplementary information

Supplementary Figure S1

Supplementary Table S1

Supplementary Table S2

Supplementary Table S3

Supplementary Table S4

Supplementary Table S5

Supplementary Table S6

Supplementary Table S7

Supplementary Table S8

## References

[1] Ferlay J, Soerjomataram I, Dikshit R, Eser S, Mathers C, Rebelo M (2015). Cancer incidence and mortality worldwide: sources, methods and major patterns in GLOBOCAN 2012. Int J Cancer.

[2] Wu WKK, Cho CH, Lee CW, Fan D, Wu K, Yu J (2010). Dysregulation of cellular signaling in gastric cancer. Cancer Lett.

[3] Polkowski W, van Sandick JW, Offerhaus GJA, ten Kate FJW, Mulder J, Obertop H (1999). Prognostic value of Lauren classification and c-erbB-2 oncogene overexpression in adenocarcinoma of the esophagus and gastroesophageal junction. Ann Surg Oncol.

[4] Cancer Genome Atlas Research N. (2014). Comprehensive molecular characterization of gastric adenocarcinoma. Nature.

[5] Molaei F, Forghanifard MM, Fahim Y, Abbaszadegan MR (2018). Molecular signaling in tumorigenesis of gastric cancer. Iran Biomed J.

[6] Andersson ER, Lendahl U (2014). Therapeutic modulation of Notch signalling—are we there yet?. Nat Rev Drug Discov.

[7] Kopan R, Ilagan MXG (2009). The canonical Notch signaling pathway: unfolding the activation mechanism. Cell.

[8] Yeh T-S, Wu C-W, Hsu K-W, Liao W-J, Yang M-C, Li AF-Y (2009). The activated Notch1 signal pathway is associated with gastric cancer progression through cyclooxygenase-2. Cancer Res.

[9] Kim SJ, Lee HW, Baek JH, Cho YH, Kang HG, Jeong JS (2016). Activation of nuclear PTEN by inhibition of Notch signaling induces G2/M cell cycle arrest in gastric cancer. Oncogene.

[10] Lu J, Getz G, Miska EA, Alvarez-Saavedra E, Lamb J, Peck D (2005). MicroRNA expression profiles classify human cancers. Nature.

[11] Jansson MD, Lund AH (2012). MicroRNA and cancer. Mol Oncol.

[12] Garzon R, Marcucci G, Croce CM (2010). Targeting microRNAs in cancer: rationale, strategies and challenges. Nat Rev Drug Discov.

[13] Li Z, Rana TM (2014). Therapeutic targeting of microRNAs: current status and future challenges. Nat Rev Drug Discov.

[14] Gao J, Aksoy BA, Dogrusoz U, Dresdner G, Gross B, Sumer SO (2013). Integrative analysis of complex cancer genomics and clinical profiles using the cBioPortal. Sci Signal.

[15] Qian Z, Zhu G, Tang L, Wang M, Zhang L, Fu J (2014). Whole genome gene copy number profiling of gastric cancer identifies PAK1 and KRAS gene amplification as therapy targets. Genes Chromosomes Cancer.

[16] He PY, Yip WK, Chai BL, Chai BY, Jabar MF, Dusa N (2017). Inhibition of cell migration and invasion by miR‑29a‑3p in a colorectal cancer cell line through suppression of CDC42BPA mRNA expression. Oncol Rep.

[17] Chen G, Zhou T, Li Y, Yu Z, Sun L (2017). p53 target miR-29c-3p suppresses colon cancer cell invasion and migration through inhibition of PHLDB2. Biochem Biophys Res Commun.

[18] Zhou QL, Jiang ZY, Mabardy AS, Del Campo CM, Lambright DG, Holik J (2010). A novel pleckstrin homology domain-containing protein enhances insulin-stimulated Akt phosphorylation and GLUT4 translocation in adipocytes. J Biol Chem.

[19] Radtke F, Raj K (2003). The role of Notch in tumorigenesis: oncogene or tumour suppressor?. Nat Rev Cancer.

[20] Capaccione KM, Pine SR (2013). The Notch signaling pathway as a mediator of tumor survival. Carcinogenesis.

[21] Mathé EA, Nguyen GH, Bowman ED, Zhao Y, Budhu A, Schetter AJ (2009). MicroRNA expression in squamous cell carcinoma and adenocarcinoma of the esophagus: associations with survival. Clin Cancer Res.

[22] Rangarajan A, Talora C, Okuyama R, Nicolas M, Mammucari C, Oh H (2001). Notch signaling is a direct determinant of keratinocyte growth arrest and entry into differentiation. EMBO J.

[23] Ronchini C, Capobianco AJ (2001). Induction of cyclin D1 transcription and CDK2 activity by Notchic: implication for cell cycle disruption in transformation by Notchic. Mol Cell Biol.

[24] Vilimas T, Mascarenhas J, Palomero T, Mandal M, Buonamici S, Meng F (2007). Targeting the NF-κB signaling pathway in Notch1-induced T-cell leukemia. Nat Med.

[25] Weng AP, Millholland JM, Yashiro-Ohtani Y, Arcangeli ML, Lau A, Wai C (2006). c-Myc is an important direct target of Notch1 in T-cell acute lymphoblastic leukemia/lymphoma. Genes Dev.

[26] Stylianou S, Clarke RB, Brennan K (2006). Aberrant activation of notch signaling in human breast cancer. Cancer Res.

[27] Fernandez-Majada V, Aguilera C, Villanueva A, Vilardell F, Robert-Moreno A, Aytes A (2007). Nuclear IKK activity leads to dysregulated notch-dependent gene expression in colorectal cancer. Proc Natl Acad Sci.

[28] Kimura K, Satoh K, Kanno A, Hamada S, Hirota M, Endoh M (2007). Activation of Notch signaling in tumorigenesis of experimental pancreatic cancer induced by dimethylbenzanthracene in mice. Cancer Sci.

[29] Serafin V, Persano L, Moserle L, Esposito G, Ghisi M, Curtarello M (2011). Notch3 signalling promotes tumour growth in colorectal cancer. J Pathol.

[30] Ozawa T, Kazama S, Akiyoshi T, Murono K, Yoneyama S, Tanaka T (2014). Nuclear Notch3 expression is associated with tumor recurrence in patients with stage II and III colorectal cancer. Ann Surg Oncol.

[31] Pastò A, Serafin V, Pilotto G, Lago C, Bellio C, Trusolino L (2014). NOTCH3 signaling regulates MUSASHI-1 expression in metastatic colorectal cancer cells. Cancer Res.

[32] Nguyen B-C, Lefort K, Mandinova A, Antonini D, Devgan V, Della Gatta G (2006). Cross-regulation between Notch and p63 in keratinocyte commitment to differentiation. Genes Dev.

[33] Pine SR, Marshall B, Varticovski L (2008). Lung cancer stem cells. Dis mark.

[34] Zhang H, Wang X, Xu J, Sun Y (2014). Notch1 activation is a poor prognostic factor in patients with gastric cancer. Br J Cancer.

[35] Piazzi G, Bazzoli F, Ricciardiello L (2012). Epigenetic silencing of Notch signaling in gastrointestinal cancers. Cell Cycle.

[36] Hsu K-W, Hsieh R-H, Huang K-H, Li AF-Y, Chi C-W, Wang T-Y (2012). Activation of the Notch1/STAT3/Twist signaling axis promotes gastric cancer progression. Carcinogenesis.

[37] Bauer L, Langer R, Becker K, Hapfelmeier A, Ott K, Novotny A (2012). Expression profiling of stem cell-related genes in neoadjuvant-treated gastric cancer: a NOTCH2, GSK3B and β-catenin gene signature predicts survival. Plos ONE.

[38] Huang T-T, Ping Y-H, Wang A-M, Ke C-C, Fang W-L, Huang K-H (2015). The reciprocal regulation loop of Notch2 pathway and miR-23b in controlling gastric carcinogenesis. Oncotarget.

[39] Guo L-Y, Li Y-M, Qiao L, Liu T, Du Y-Y, Zhang J-Q (2012). Notch2 regulates matrix metallopeptidase 9 via PI3K/AKT signaling in human gastric carcinoma cell MKN-45. World J Gastroenterol.

[40] Kang H, An H-J, Song J-Y, Kim T-H, Heo J-H, Ahn D-H (2012). Notch3 and Jagged2 contribute to gastric cancer development and to glandular differentiation associated with MUC2 and MUC5AC expression. Histopathology.

[41] Qian C, Liu F, Ye B, Zhang X, Liang Y, Yao J (2015). Notch4 promotes gastric cancer growth through activation of Wnt1/β-catenin signaling. Mol Cell Biochem.

[42] Denoyelle C, Lambert B, Meryet-Figuiere M, Vigneron N, Brotin E, Lecerf C (2014). miR-491-5p-induced apoptosis in ovarian carcinoma depends on the direct inhibition of both BCL-X L and EGFR leading to BIM activation. Cell Death Dis.

[43] Gong F, Ren P, Zhang Y, Jiang J, Zhang H (2016). MicroRNAs-491-5p suppresses cell proliferation and invasion by inhibiting IGF2BP1 in non-small cell lung cancer. Am J Transl Res.

[44] Zhao Q, Zhai Y-X, Liu H-Q, Shi Y-A, Li X-B (2015). MicroRNA-491-5p suppresses cervical cancer cell growth by targeting hTERT. Oncol Rep.

[45] Zhang T, Cai X, Li Q, Xue P, Chen Z, Dong X (2016). Hsa-miR-875-5p exerts tumor suppressor function through down-regulation of EGFR in colorectal carcinoma (CRC). Oncotarget.

[46] Takebe N, Nguyen D, Yang SX (2014). Targeting notch signaling pathway in cancer: clinical development advances and challenges. Pharmacol Therap.

[47] Yuan X, Wu H, Xu H, Xiong H, Chu Q, Yu S (2015). Notch signaling: an emerging therapeutic target for cancer treatment. Cancer Lett.

[48] Strosberg JR, Yeatman T, Weber J, Coppola D, Schell MJ, Han G (2012). A phase II study of RO4929097 in metastatic colorectal cancer. Eur J Cancer.

[49] Tolcher AW, Messersmith WA, Mikulski SM, Papadopoulos KP, Kwak EL, Gibbon DG (2012). Phase I study of RO4929097, a gamma secretase inhibitor of Notch signaling, in patients with refractory metastatic or locally advanced solid tumors. J Clin Oncol.

[50] Nair JS, Sheikh T, Ho AL, Schwartz GK (2013). PTEN regulates sensitivity of melanoma cells to RO4929097, the gamma-secretase inhibitor. Anticancer Res.

[51] De Jesus-Acosta A, Laheru D, Maitra A, Arcaroli J, Rudek MA, Dasari A (2014). A phase II study of the gamma secretase inhibitor RO4929097 in patients with previously treated metastatic pancreatic adenocarcinoma. Investig N. Drugs.

[52] Luistro L, He W, Smith M, Packman K, Vilenchik M, Carvajal D (2009). Preclinical profile of a potent gamma-secretase inhibitor targeting notch signaling with in vivo efficacy and pharmacodynamic properties. Cancer Res.

[53] Bellacosa A, Testa JR, Staal SP, Tsichlis PN (1991). A retroviral oncogene, akt, encoding a serine-threonine kinase containing an SH2-like region. Science.

[54] Datta SR, Brunet A, Greenberg ME (1999). Cellular survival: a play in three Akts. Genes Dev.

[55] Liu LZ, Zhou XD, Qian G, Shi X, Fang J, Jiang BH (2007). AKT1 amplification regulates cisplatin resistance in human lung cancer cells through the mammalian target of rapamycin/p70S6K1 pathway. Cancer Res.

[56] Hirai H, Sootome H, Nakatsuru Y, Miyama K, Taguchi S, Tsujioka K (2010). MK-2206, an allosteric Akt inhibitor, enhances antitumor efficacy by standard chemotherapeutic agents or molecular targeted drugs in vitro and in vivo. Mol Cancer Ther.

[57] Ji D, Zhang Z, Cheng L, Chang J, Wang S, Zheng B (2014). The combination of RAD001 and MK-2206 exerts synergistic cytotoxic effects against PTEN mutant gastric cancer cells: involvement of MAPK-dependent autophagic, but not apoptotic cell death pathway. Plos ONE.

[58] Kang W, Tong JHM, Chan AWH, Lung RWM, Chau SL, Wong QWL (2012). Stathmin1 plays oncogenic role and is a target of microRNA-223 in gastric cancer. Plos ONE.

[59] Kang W, Tong JH, Chan AW, Lee TL, Lung RW, Leung PP (2011). Yes-associated protein 1 exhibits oncogenic property in gastric cancer and its nuclear accumulation associates with poor prognosis. Clin Cancer Res.

