# Supplementary figures and images for: NOTCH3, a crucial target of miR-491-5p/miR-875-5p, promotes gastric carcinogenesis by upregulating PHLDB2 expression and activating Akt pathway
Source: Oncogene. 2021 Jan 15;40(9):1578–94. doi: 10.1038/s41388-020-01579-3 (PMC7932926; doi:10.1038/s41388-020-01579-3)

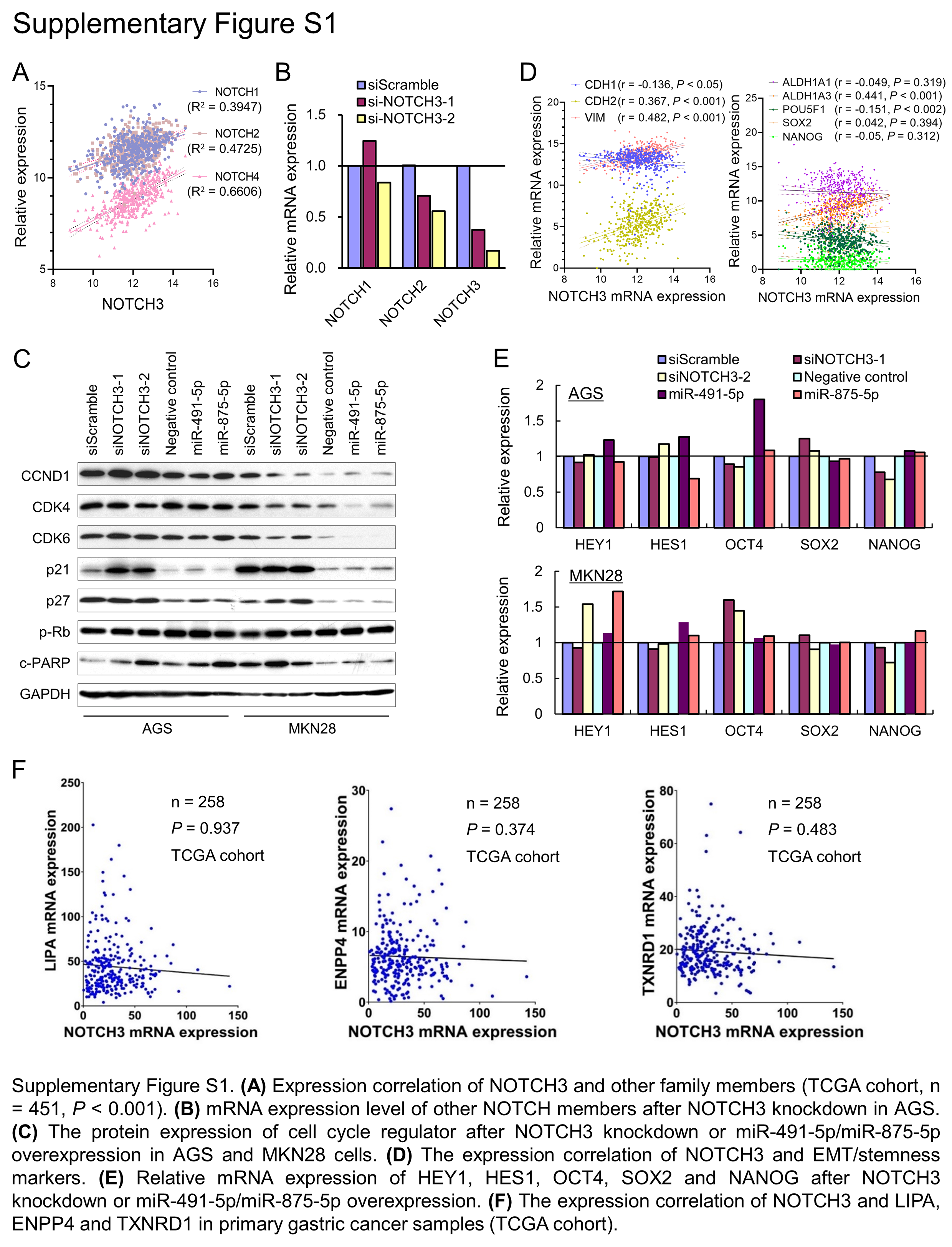

Supplement: Supplementary file 1 — Supplementary Figure S1 [file 41388_2020_1579_MOESM1_ESM.tif]
