# Supplementary material for: NOTCH3, a crucial target of miR-491-5p/miR-875-5p, promotes gastric carcinogenesis by upregulating PHLDB2 expression and activating Akt pathway
Source: Oncogene. 2021 Jan 15;40(9):1578–94. doi: 10.1038/s41388-020-01579-3 (PMC7932926; doi:10.1038/s41388-020-01579-3)
Supplement: Supplementary file 2 — Supplementary Table S1 [file 41388_2020_1579_MOESM2_ESM.doc]

**Supplementary Table S1 Correlation of NOTCH3 nuclear accumulation in GC with other clinicopathologic features (significant *P*-value in bold and Italic format). The case number and percentage counted were shown in the table.**

|  |  | Gastric cancer (n = 273) | | | |
| --- | --- | --- | --- | --- | --- |
|  |  | Negative/weak | | Moderate/strong | *P*-value |
| Sex | M | | 88 (48.1%) | 95 (51.9%) | 0.107 |
|  | F | | 34 (37.8%) | 56 (62.2%) |  |
| Age | <=60 | | 49 (47.6%) | 54 (52.4%) | 0.456 |
|  | >60 | | 73 (42.9%) | 97 (57.1%) |  |
| Type | Intestinal | | 68 (46.9%) | 77 (53.1%) | 0.469 |
|  | Diffuse | | 54 (42.5%) | 73 (57.5%) |  |
| Grade | 1 | | 6 (66.7%) | 3 (33.3%) | 0.386 |
|  | 2 | | 45 (45.0%) | 55 (55.0%) |  |
|  | 3 | | 70 (43.2%) | 92 (56.8%) |  |
| Stage | 1 | | 26 (44.1%) | 33 (55.9%) | 0.681 |
|  | 2 | | 14 (43.8%) | 18 (56.2%) |  |
|  | 3 | | 43 (50.0%) | 43 (50.0%) |  |
|  | 4 | | 39 (41.1%) | 56 (58.9%) |  |
| Stage (T) | 1 | | 11 (29.7%) | 26 (70.3%) | 0.252 |
|  | 2 | | 37 (48.1%) | 40 (51.9%) |  |
|  | 3 | | 68 (47.2%) | 76 (52.8%) |  |
|  | 4 | | 6 (42.9%) | 8 (57.1%) |  |
| Stage (N) | 0 | | 28 (46.7%) | 32 (53.3%) | 0.477 |
|  | 1 | | 33 (47.1%) | 37 (52.9%) |  |
|  | 2 | | 39 (48.1%) | 42 (51.9%) |  |
|  | 3 | | 22 (36.1%) | 39 (63.9%) |  |
| Stage (M) | 0 | | 99 (43.0%) | 131 (57.0%) | 0.160 |
|  | 1 | | 23 (54.8%) | 19 (45.2%) |  |
| Lymph Node | 0 | | 28 (46.7%) | 32 (53.3%) | 0.749 |
|  | 1 | | 94 (44.3%) | 118 (55.7%) |  |
| *H. pylori* | Absence | | 55 (45.1%) | 67 (54.9%) | 0.834 |
|  | Presence | | 64 (46.4%) | 74 (53.6%) |  |
