# Supplementary material for: NOTCH3, a crucial target of miR-491-5p/miR-875-5p, promotes gastric carcinogenesis by upregulating PHLDB2 expression and activating Akt pathway
Source: Oncogene. 2021 Jan 15;40(9):1578–94. doi: 10.1038/s41388-020-01579-3 (PMC7932926; doi:10.1038/s41388-020-01579-3)
Supplement: Supplementary file 3 — Supplementary Table S2 [file 41388_2020_1579_MOESM3_ESM.doc]

**Supplementary Table S2** Univariate and multivariate Cox regression analysis of the association between clinicopathologic characteristics and disease specific survival in patients with gastric adenocarcinoma (n = 255, significant *P*-value in bold and Italic format).

|  | Univariate analysis | Multivariate analysis |
| --- | --- | --- |
| Sex | 0.285 |  |
| Age | ***0.042*** | ***<0.001*** |
| Type | ***<0.001*** | 0.809 |
| Grade | ***0.006*** | 0.878 |
| Stage | ***<0.001*** | 0.162 |
| Stage (T) | ***<0.001*** | ***0.007*** |
| Stage (N) | ***<0.001*** | ***0.010*** |
| Stage (M) | ***<0.001*** | ***<0.001*** |
| Lymph Node | ***<0.001*** | 0.894 |
| *H. pylori* | 0.210 |  |
| NOTCH3 | ***0.002*** | ***0.005*** |
