# Supplementary material for: NOTCH3, a crucial target of miR-491-5p/miR-875-5p, promotes gastric carcinogenesis by upregulating PHLDB2 expression and activating Akt pathway
Source: Oncogene. 2021 Jan 15;40(9):1578–94. doi: 10.1038/s41388-020-01579-3 (PMC7932926; doi:10.1038/s41388-020-01579-3)
Supplement: Supplementary file 4 — Supplementary Table S3 [file 41388_2020_1579_MOESM4_ESM.doc]

**Supplementary Table S3** List of the top miRNAs putatively targeting NOTCH3 predicted by microRNA.org (target sites of conserved miRNA with good mirSVR scores).

| miRNA | mirSVR score | Also predicted by TargetScan |
| --- | --- | --- |
| miR-875-5p | -0.8539 | Yes |
| miR-491-5p | -0.5505/-0.1417 | Yes |
| miR-7 | -0.5677 | Yes |
| miR-186 | -0.2312 | No |
| miR-185 | -0.4185 | Yes |
| miR-300 | -0.2914 | Yes |
| miR-381 | -0.2914 | No |
| miR-485-5p | -0.2728 | Yes |
| miR-874 | -0.2154 | Yes |
| miR-296-3p | -0.1796 | Yes |
| miR-136 | -0.1330 | Yes |
| miR-31 | -0.1243 | No |
