# Supplementary material for: NOTCH3, a crucial target of miR-491-5p/miR-875-5p, promotes gastric carcinogenesis by upregulating PHLDB2 expression and activating Akt pathway
Source: Oncogene. 2021 Jan 15;40(9):1578–94. doi: 10.1038/s41388-020-01579-3 (PMC7932926; doi:10.1038/s41388-020-01579-3)
Supplement: Supplementary file 6 — Supplementary Table S5 [file 41388_2020_1579_MOESM6_ESM.doc]

|  |  | Gastric cancer (n = 252) | | |
| --- | --- | --- | --- | --- |
|  |  | Negative/weak | Moderate/strong | *P*-value |
| Sex | M | 93 (55.0%) | 76 (45.0%) | 0.903 |
|  | F | 45 (54.2%) | 38 (45.8%) |  |
| Age | <=60 | 60 (65.2%) | 32 (34.8%) | ***0.011*** |
|  | >60 | 78 (48.8%) | 82 (51.2%) |  |
| Type | Intestinal | 73 (52.9%) | 65 (47.1%) | 0.554 |
|  | Diffuse | 64 (56.6%) | 49 (43.4%) |  |
| Grade | 1 | 3 (42.9%) | 4 (57.1%) | 0.492 |
|  | 2 | 56 (58.9%) | 39 (41.1%) |  |
|  | 3 | 78 (52.3%) | 71 (47.7%) |  |
| Stage | 1 | 29 (54.7%) | 24 (45.3%) | 0.459 |
|  | 2 | 21 (67.7%) | 10 (32.3%) |  |
|  | 3 | 44 (51.8%) | 41 (48.2%) |  |
|  | 4 | 43 (52.4%) | 39 (47.6%) |  |
| Stage (T) | 1 | 18 (54.5%) | 15 (45.5%) | 0.726 |
|  | 2 | 42 (58.3%) | 30 (41.7%) |  |
|  | 3 | 73 (53.7%) | 63 (46.3%) |  |
|  | 4 | 4 (40.0%) | 6 (60.0%) |  |
| Stage (N) | 0 | 35 (66.0%) | 18 (34.0%) | 0.270 |
|  | 1 | 38 (54.3%) | 32 (45.7%) |  |
|  | 2 | 37 (49.3%) | 38 (50.7%) |  |
|  | 3 | 27 (50.9%) | 26 (49.1%) |  |
| Stage (M) | 0 | 117 (54.4%) | 98 (45.6%) | 0.899 |
|  | 1 | 20 (55.6%) | 16 (44.4%) |  |
| Lymph Node | 0 | 35 (66.0%) | 18 (34.0%) | 0.059 |
|  | 1 | 102 (51.5%) | 96 (48.5%) |  |
| *H. pylori* | Absence | 55 (48.7%) | 58 (51.3%) | 0.056 |
|  | Presence | 78 (60.9%) | 50 (39.1%) |  |

**Supplementary Table S5** Correlation of PHLDB2 cytoplasmic expression in GC with other clinicopathologic features (significant *P*-value in bold and Italic format). The case number and percentage counted were shown in the table.
