# Supplementary material for: NOTCH3, a crucial target of miR-491-5p/miR-875-5p, promotes gastric carcinogenesis by upregulating PHLDB2 expression and activating Akt pathway
Source: Oncogene. 2021 Jan 15;40(9):1578–94. doi: 10.1038/s41388-020-01579-3 (PMC7932926; doi:10.1038/s41388-020-01579-3)
Supplement: Supplementary file 7 — Supplementary Table S6 [file 41388_2020_1579_MOESM7_ESM.doc]

**Supplementary Table S6 Univariate and multivariate Cox regression analysis of the association between clinicopathologic characteristics and disease specific survival in patients with gastric adenocarcinoma (n = 255, significant *P*-value in bold and Italic format).**

|  | Univariate analysis | Multivariate analysis |
| --- | --- | --- |
| Sex | 0.286 |  |
| Age | ***0.043*** | **<*0.001*** |
| Type | ***<0.001*** | 0.828 |
| Grade | ***0.006*** | 0.810 |
| Stage | ***<0.001*** | 0.066 |
| Stage (T) | ***<0.001*** | ***0.044*** |
| Stage (N) | ***<0.001*** | 0.092 |
| Stage (M) | ***<0.001*** | ***0.007*** |
| Lymph Node | ***<0.001*** | 0.986 |
| *H. pylori* | 0.211 |  |
| PHLDB2 | ***0.006*** | 0.194 |
