# Supplementary material for: NOTCH3, a crucial target of miR-491-5p/miR-875-5p, promotes gastric carcinogenesis by upregulating PHLDB2 expression and activating Akt pathway
Source: Oncogene. 2021 Jan 15;40(9):1578–94. doi: 10.1038/s41388-020-01579-3 (PMC7932926; doi:10.1038/s41388-020-01579-3)
Supplement: Supplementary file 8 — Supplementary Table S7 [file 41388_2020_1579_MOESM8_ESM.doc]

**Supplementary Table S7** **Primers used in qRT-PCR for this study.**

| Primer | Sense (5’-3’) | Antisense (5’-3’) |
| --- | --- | --- |
| NOTCH1 | ACTGTGAGGACCTGGTGGAC | TTGTAGGTGTTGGGGAGGTC |
| NOTCH2 | TGTGACATAGCAGCCTCCAG | CAGGGGGCACTGACAGTAAT |
| NOTCH3 | GTCGTGGCTACACTGGACCT | AATGTCCACCTCGCAATAGG |
| NOTCH4 | CACGTGAACCCATGTGAGTC | TTGAGCAGTTCTGTCCATCG |
| HEY1 | GCCCTTGCTATGGACTATCG | TTGTTGAGATGCGAAACCAG |
| HES1 | TCAACACGACACCGGATAAA | TCAGCTGGCTCAGACTTTCA |
| OCT4 | GAAGGATGTGGTCCGAGTGT | GTGAAGTGAGGGCTCCCATA |
| SOX2 | ACACCAATCCCATCCACACT | TTTTTCGTCGCTTGGAGACT |
| NANOG | GATTTGTGGGCCTGAAGAAA | AAGTGGGTTGTTTGCCTTTG |
| B2M | ACTCTCTCTTTCTGGCCTGG | ATGTCGGATGGATGAAACCC |
