# Supplementary material for: NOTCH3, a crucial target of miR-491-5p/miR-875-5p, promotes gastric carcinogenesis by upregulating PHLDB2 expression and activating Akt pathway
Source: Oncogene. 2021 Jan 15;40(9):1578–94. doi: 10.1038/s41388-020-01579-3 (PMC7932926; doi:10.1038/s41388-020-01579-3)
Supplement: Supplementary file 9 — Supplementary Table S8 [file 41388_2020_1579_MOESM9_ESM.doc]

**Supplementary Table S8** Oligonucleotides used in the luciferase activity experiments. The oligonucleotides were annealed and subcloned into pMIR-REPORT via *HindIII* and *SpeI* restriction sites. WT (Wild type), full length of the putative miRNA binding site; Mutation, the binding site was mutated.

| Oligonucleotides | Sequence (5’-3’) |
| --- | --- |
| miR-491-5p_WT (BS1)_Sense | CTAGTTGGGACCTCCTTCTTCCCCACTA |
| miR-491-5p _WT (BS1)_Antisense | AGCTTAGTGGGGAAGAAGGAGGTCCCAA |
| miR-491-5p _ Mutation (BS1)_Sense | CTAGTTGGGACCTCCTTCAAGGGGTGTA |
| miR-491-5p _Mutation (BS1)_ Antisense | AGCTTACACCCCTTGAAGGAGGTCCCAA |
| miR-491-5p _WT (BS2)_Sense | CTAGTAGTGTTGGGAGCCTCCTCCCCACCA |
| miR-491-5p _WT (BS2)_Antisense | AGCTTGGTGGGGAGGAGGCTCCCAACACTA |
| miR-491-5p _Mutation (BS2)_Sense | CTAGTAGTGTACCGTCCCTCCAGGGGTGGA |
| miR-491-5p _Mutation (BS2)_ Antisense | AGCTTCCACCCCTGGAGGGACGGTACACTA |
| miR-875-5p_WT _Sense | CTAGTCTCATGGCAGAATAGAGGTATTA |
| miR-875-5p _WT _Antisense | AGCTTAATACCTCTATTCTGCCATGAGA |
| miR-875-5p _ Mutation _Sense | CTAGTCTCATGGCAGAATACTCCATATA |
| miR-875-5p _Mutation _ Antisense | AGCTTATATGGAGTATTCTGCCATGAGA |
